# Association between Pregnancy and Active Injection Drug Use and Sex Work among Women Injection Drug Users in Saint Petersburg, Russia

**DOI:** 10.1007/s11524-015-9954-3

**Published:** 2015-04-03

**Authors:** P. Girchenko, D. C. Ompad, D. Bikmukhametov, L. Gensburg

**Affiliations:** 1 Department of Epidemiology and Biostatistics, School of Public Health, University at Albany, State University of New York, Rensselaer, NY USA; 2 Global Institute of Public Health, New York University, New York, NY USA; 3 Center for Drug Use and HIV Research, New York University, New York, NY USA; 4 Center for Health, Identity, Behavior, and Prevention Studies, New York University, New York, NY USA; 5 German Doctor Exchange, Bonn, Germany

**Keywords:** Pregnancy, Injection drug use, Sex work in St. Petersburg

## Abstract

Widespread use of unsafe sexual practices among women injecting drugs both practicing and not practicing sex work leads to high levels of unplanned pregnancies in this population. The goal of this study was to investigate the association between pregnancy and active drug use and sex work. Data were collected using a convenience sample of 500 women in Saint Petersburg, Russia, in 2013. All women had recent experience of drug use, of which 200 were pregnant at the time of the study. The study consisted of a structured interview followed by a rapid HIV test. Pregnancy was protective against both active drug use and sex work. For HIV-positive women, these associations were stronger than for HIV-negative women: drug use prevalence ratio (PR) was 0.59 vs 0.85; for sex work, the PRs were 0.36 vs 0.64. Higher levels of education were associated with a lower prevalence ratio for active drug use and sex work in all models. Having children was not associated with active drug use or sex work. Pregnancy might be an optimal time for conducting interventions aimed at cessation of drug use and sex work among women injecting drugs.

## Introduction

The HIV epidemic in the Russian Federation continues to remain one of the fastest growing HIV epidemics worldwide.^1^ As of the end of 2013, HIV prevalence in the Russian Federation was 468.8 per 100,000 of population; in total, 800,531 people were registered with HIV.^2^ Those officially registered with HIV infection are patients who were non-anonymously tested at the state medical facility, tested positive on the enzyme-linked immunosorbent assay (ELISA) test, and confirmed by the Western blot. It is suggested that official data seriously underestimate the real HIV prevalence in the Russian Federation.^3^^,^ 4

From the beginning of the HIV epidemic in the Russian Federation, injection drug users (IDUs) were the most affected group with the main route of HIV transmission through infected syringes.^5^ Presently, heterosexual HIV transmission is increasing: 42 % of 79,728 people diagnosed with HIV in 2013 in the Russian Federation were infected through heterosexual sex.^2^ However, IDUs continue to comprise a substantial proportion of people infected with HIV.^6^

Previous research demonstrates that, among women, the use of illegal drugs and unsafe sexual practices are correlated. Some studies suggest that around 40 % of women IDUs (WIDUs) are involved in transactional sex or commercial sex work^7^^,^ 8 and between 20 and 80 % of the women engaged in sex work also inject drugs.^9^ Because of sex work and unsafe sexual behaviors, WIDUs may serve as a bridge of HIV transmission to the general population.^10^

Studies have shown a high prevalence of unsafe sexual practices among WIDUs. One such study’s finding was that only 22 % of WIDUs in Saint Petersburg reported consistently using condoms.^11^ This, in turn, leads to a high level of unplanned and unwanted pregnancies^11^^,^ 12 resulting in both higher levels of pregnancy terminations^13^ and increased number of births among WIDUs.^14^

Pregnancy, complicated by injection drug use and positive HIV status and/or other sexually transmitted infections, raises many public health issues. These include mother-to-child HIV and drug addiction transmissions, abandonment of infants by HIV-positive mothers,^15^ and health complications for the mother and the baby.^16^

Nevertheless, for women using drugs, pregnancy might be an optimal time for interventions aimed not only at prevention of mother-to-child HIV transmission but also at treatment of drug addiction. Massey^17^ found that women’s motivation to change their substance use behaviors during pregnancy is high; additionally, the cessation of injection drug use would not only improve the health of newborns but may also lead to safer sexual practices. These interventions might result in decreasing the levels of HIV infection and HIV transmission in the population.

The purpose of this study is to assess the associations between pregnancy and active injection drug use and active sex work in a convenience sample of women injection drug users in Russia.

## Methods

### Setting

Saint Petersburg is the second largest city in Russia with a population of 5.3 million people or 3.2 % of the total population of Russia.^18^ The estimated number of IDUs in Saint Petersburg is between 30,000 and 80,000,^19^ with the estimated HIV prevalence among IDUs which ranges between 40 and 64 %.^6^^,^ 19

There are no reliable data available on the sex distribution among IDUs in Saint Petersburg, but some data indicate that women comprise between 20 and 40 % of the total number of IDUs.^7^^,^ 11 Unpublished data collected from 1006 respondents by one of the largest harm reduction programs in Saint Petersburg (*Humanitarian Action*)^20^ suggest that the proportion of women among IDUs in Saint Petersburg might be as high as 45 %.

### Study Population

A convenience sample of women was recruited who were current or past injection drug users and had either (1) received harm reduction services at an outreach program tailored for women or (2) participated in a case management program designed to assist pregnant women using drugs. Both programs are implemented by the fund Humanitarian Action. Women who participated in these programs between May and October of 2013 were invited to participate in the study by the program’s staff. Following agreement, a trained interviewer conducted all study procedures with the exception of the blood tests which were performed by a certified nurse.

Additional women not participating in Humanitarian Action programs were recruited by word of mouth as information about the study spread through the community of female drug users in Saint Petersburg.

The inclusion criteria for the study were women aged 18 and 48 years with recent experience of injection drug use (defined as at least one injection during the previous 6 months). The lower age limit was determined by Russian regulations on performing HIV tests: only persons aged 18 and older can legally provide informed consent for HIV testing; the highest age limit was chosen to reflect the reproductive years.

Women were informed that their participation or refusal to participate in this study would not affect their receiving programmatic services from Humanitarian Action. Women who chose to participate in the study completed face-to-face interviews using a structured questionnaire and underwent rapid HIV testing (Alere Determine HIV1/HIV2; Waltham, MA, USA). Rapid testing for HIV was preceded by pre-test counseling and followed by post-test counseling according to Russian regulations. In the case of a positive test result, women were referred to a medical facility for HIV diagnosis, since according to Russian regulations, rapid tests are not diagnostic instruments and might only serve as motivators to seek medical care. Participants were offered a phone card worth $10 as an incentive. Participation in the study was completely anonymous, participants provided no identifying information: instead of a name, an individual depersonalized code was used. In addition, to provide absolute anonymity, instead of date of birth, only the year of birth was collected. Study protocol was approved by the local IRB; all women who choose to participate in the study have signed written inform consent form.

In total, of 584 women invited to participate, 534 were eligible, and of these, 500 (94 %) provided informed consent and took part in the study. Two hundred of these were pregnant at the time of their interview. Since the study goal was to assess associations between risk behaviors and pregnancy, pregnant women were oversampled: the initial goal of recruitment was set up to recruit at least 500 women of which 200 should be pregnant.

### Data Analysis

Descriptive statistics were calculated (means and medians) for continuous variables and frequencies and percentages for categorical variables. Bivariate analyses were conducted to assess differences between women who were pregnant at the time of study and those who were not, and included *t* tests for continuous variables and *χ* ^2^ square tests for categorical variables. Previous research has found prevalence ratios (PRs) to be an appropriate effect measure for cross-sectional studies^21^ in which the outcomes were common. Thus, log-binomial regression, which has been proven to calculate PR accurately,^22^ was chosen to measure the associations of interest. To assess the PR of active drug use and/or active sex work among pregnant women compared to non-pregnant women, two models were constructed using pregnancy status (yes/no) as the primary predictor variable. Additional covariates included age, education, and parental status. Age and education were found to be important confounders in other studies.^12^ Little is known about parental status as a potential confounder of the association between pregnancy and active injection drug use and/or sex work, but some evidence indicates it might be a confounder.^23^^,^ 24 All potential covariates were associated both with exposure and the outcomes in bivariate analyses and met the criteria for being potential confounders according to constructed directed acyclic graphs (DAGs). Based on DAG, HIV status did not meet the criteria for being a confounder as it is a consequence of risky behaviors; it is a surrogate for sharing needles and/or having sex without condoms in the past and thus might potentially be related to changes in drug use/sex work. Thus, it is a potential effect modifier. To assess potential differences in associations between exposure and both outcomes for HIV-positive and HIV-negative women, interaction terms between the main exposure (pregnancy) and HIV status (positive/negative) were included in the both models. Since active drug use and sex work are highly correlated, it was not feasible to include these variables as additional covariates in the corresponding models. Data were analyzed using SAS 9.3 software (SAS Institute Inc., Cary, NC, USA).

## Results

Descriptive characteristics of the sample are presented in Table 1. The mean age of the women was 31 years (range 18–47 years); 86.2 % had at least a high school education, and 13.8 % had post college education. Only 7.6 % of the women reported that they had never been pregnant during their life, 71.6 % had had at least one abortion, and 71 % had children. Forty percent were pregnant at the time of the study, and 22.3 % reported that they had realized their last or current pregnancy after 12 weeks of gestation. According to Russian regulations, 12 weeks is the cutoff point after which elective abortions cannot be performed. Approximately 75 % of the women reported active drug use, and 41.4 % said that they were actively practicing sex work at the time of the study. Rapid HIV tests were positive for 62.9 % of the study population.

**TABLE 1:** Characteristics of study population in 2013

Characteristic	Total	Pregnant	Not pregnant	*p* value
Mean age (SD), years	31.0 (4.8)	29.6	31.9	<0.001
Education, *n* (%)				
Unfinished high school	69 (13.8)	36 (12.0)	33 (16.5)	0.13
High school	210 (42.0)	120 (40.0)	90 (45.0)	
Some college	152 (30.4)	102 (34.0)	50 (25)	
Post college education	69 (13.8)	42 (14.0)	27 (13.5)	
Pregnancy at the time of interview, *n* (%)				
No	300 (60.0)			
Yes	200 (40.0)			
Ever been pregnant, *n* (%)				
No	38 (7.6)			
Yes	462 (96.4)			
Parental status, *n* (%)				
No	145 (29.0)	19 (9.5)	126 (42)	<0.001
Yes	355 (71.0)	181 (90.5)	174 (58.0)	
Ever had an abortion, *n* (%)				
No	142 (28.4)	75 (37.5)	110 (36.9)	0.92
Yes	358 (71.6)	125 (62.5)	188 (63.1)	
HIV status, *n* (%)				
Negative	184 (37.1)	46 (23.5)	138 (46)	<0.001
Positive	312 (62.9)	150 (76.5)	162 (54)	
Active drug use at the time of interview, *n* (%)				
No	126 (25.2)	86 (43)	40 (13.3)	<0.001
Yes	374 (74.8)	114 (57)	260 (86.7)	
Active sex work at the time of interview, *n* (%)				
No	293 (58.6)	152 (76)	141 (47)	<0.001
Yes	207 (41.4)	48 (24)	159 (53)	
Discovered current/last pregnancy after 12 weeks, *n* (%)				
No	358 (77.7)	140 (70.3)	218 (83.3)	<0.001
Yes	103 (22.3)	59 (29.5)	44 (16.8)	

The proportion of HIV-positive results was significantly higher among the pregnant women than among women who were not pregnant at the time of study: 76.5 vs 54 % (*p* < 0.0001, Table 1). On average, women who were pregnant at the time of study were younger, more likely to already have children and be HIV positive, and less likely to be actively using drugs and engaging in sex work. Women who were pregnant at the time of study discovered their current pregnancy later than women who were not pregnant at the time of study and discovered their last pregnancy (*p* = 0.009): 29.7 % of women who were pregnant at the time of study discovered their pregnancy after 12 weeks vs 16.8 % of non-pregnant women who discovered their last pregnancy after 12 weeks.

Engagement in active sex work was associated with a lower proportion of HIV-positive test results (54.5 vs 68.6 %, *p* = 0.002).

### Multivariate Analysis

#### Active Drug Use

To assess potential effect modification by HIV status, the model was run with an interaction term for pregnancy status and HIV status. Since HIV status was shown to modify the association between pregnancy and drug use (interactive term: *p* = 0.003), analysis was stratified on HIV status.

The prevalence ratio of active drug use among pregnant HIV-positive WIDU was lower compared to non-pregnant HIV-positive WIDU (PR = 0.59). Although a higher level of education was shown to be protective against active drug use during pregnancy compared to high school level, it did not confound the association between pregnancy and active drug use. Age and parental status were not found to be confounders. Thus, age and parental status were not included in the final model (Table 2).

**TABLE 2:** Prevalence ratio of active drug use in pregnant HIV-positive WIDUs compared to non-pregnant HIV-positive WIDUs (*N* = 312)

	Crude PR (95 % CI)	Adjusted^a^ PR (95 % CI)
Pregnancy status	0.59 (0.50, 0.70)	0.60 (0.51, 0.71)
Education		
Unfinished high school		1.01 (0.88, 1.15)
High school		1.0
Some college		0.88 (0.75, 1.02)
Post college		0.72 (0.52, 0.99)

^a^Adjusted for education

For HIV-negative WIDU, the association between pregnancy and active drug use was found to be weaker than for HIV-positive women, but still statistically significant after adjusting for education (PR = 0.85). A higher level of education was also found to be protective against active drug use during pregnancy comparing to high school level but was not a confounder of the association between pregnancy and active drug use (Table 3). Overall, a protective effect of pregnancy against active drug use was seen among both HIV-positive and HIV-negative women; the effect was more pronounced in HIV-positive women.

**TABLE 3:** Prevalence ratio of active drug use in pregnant HIV-negative WIDUs compared to non-pregnant HIV-negative WIDUs (*N* = 184)

	Crude PR (95 % CI)	Adjusted^a^ PR (95 % CI)
Pregnancy status	0.85 (0.72, 1.01)	0.85 (0.72, 1.0)
Education		
Unfinished high school		0.99 (0.88, 1.11)
High school		1.0
Some college		0.94 (0.84, 1.05)
Post college		0.73 (0.58, 0.93)

^a^Adjusted for education

#### Active Sex Work

As with the drug use models, an interactive term with pregnancy and HIV status was included to test for effect modification. The interaction term for HIV status and pregnancy was found to be statistically significant (*p* = 0.042); thus, stratified models were run for HIV-positive and HIV-negative women (Tables 4 and 5). Age and parental status were not statistically significant and did not confound the association of interest. Although a higher level of education appears to be a protective factor against active sex work during pregnancy both in HIV-positive and HIV-negative women, it does not confound the association between pregnancy and active sex work.

**TABLE 4:** Prevalence ratio of active sex work in HIV-positive pregnant WIDUs compared to non-pregnant WIDUs (*N* = 312)

	Crude OR (95 % CI)	Adjusted^a^ PR (95 % CI)
Pregnancy status	0.36 (0.25, 0.53)	0.36 (0.25, 0.52)
Education		
Unfinished high school		1.12 (0.83, 1.52)
High school		1.0
Some college		0.60 (0.42, 0.86)
Post college		0.35 (0.15, 0.79)

^a^Adjusted for education

**TABLE 5:** Prevalence ratio of active sex work in pregnant HIV-negative WIDUs compared to non-pregnant HIV-negative WIDUs (*N* = 184)

	Crude PR (95 % CI)	Adjusted^a^ PR (95 % CI)
Pregnancy status	0.63 (0.42, 0.96)	0.64 (0.42, 0.96)
Education		
Unfinished high school		1.03 (0.72, 1.47)
High school		1.0
Some college		0.65 (0.46, 0.92)
Post college		0.61 (0.38, 0.98)

^a^Adjusted for education

The negative association between pregnancy status and active sex work was stronger than the negative association between pregnancy status and active sex drug use. For HIV-positive WIDU, this association was stronger than for HIV-negative WIDU (PR = 0.36 vs PR = 0.64).

## Discussion

Our study found pregnancy to be inversely associated with active drug use, and for HIV-positive women, this association was stronger. Similarly, a stronger inverse association between pregnancy and active sex work was found, with HIV-positive women almost two times less likely to actively practice sex work comparing to HIV-negative WIDU. Higher levels of education were found to be protective against both active drug use and active sex work during pregnancy, and it played a more significant role as a protective factor against sex work than drug use among pregnant WIDU. However, having prior children was not associated with either active drug use or active sex work among both HIV-positive and HIV-negative women. One explanation for this might be that WIDU is motivated to stop risky behaviors when they are pregnant, but the period of motivation to discontinue drug use and sex work might be limited to the period of pregnancy. This explanation is supported by findings from another study where pregnancy was associated with increased motivation to decrease drug use.^23^

Women who were pregnant at the time of study had significantly higher prevalence of HIV infection, discovered their current pregnancy later than non-pregnant women discovered their last pregnancy, and were more likely to already have children. This suggests that women who are engaged in unsafe sexual practices are more likely both to be HIV infected and pregnant. In contrast, women who reported actively practicing safe sex work were less likely both to be HIV positive and pregnant. This is in concordance with findings from other studies where practicing sex work was associated with lower levels of unsafe sex behaviors and higher level of condom use among WIDUs.^25^ These finding also suggest that interventions aimed at decreasing risky sexual behaviors would be beneficial both for addressing HIV spread and preventing unplanned and unwanted pregnancies in the population of WIDU.

Prevalence estimation of HIV infection in the study population (63 %) may not adequately reflect the prevalence of HIV in the total population of WIDU; nevertheless, this finding is alarming and indicating a serious problem concerning the spread of HIV among IDUs and WIDUs, which agrees findings of other studies.^26^^,^ 27 In 2008, it was estimated that HIV seroprevalence among injection drug users in the Russian Federation was 37.2 %.^3^ Findings from the present study might indicate that among female injection drug users, this number might have significantly increased during the last 5 years. Policies aimed at criminalization of drug use and sex work in Russia have not only led to further marginalization of populations involved in these activities but also created barriers in accessing HIV care facilities.^28^ These factors, combined with the absence of stable systematic prevention programs proven to be effective in stopping HIV epidemic in vulnerable populations, have resulted in the continuous growth of HIV infection among these groups.

Taking into account the severely high level of HIV seroprevalence among pregnant WIDUs in our study, it is not surprising that Saint Petersburg experiences the highest number of deliveries in Russia given by HIV-positive mothers.^14^

### Limitations

As with all cross-sectional studies, the temporality of exposure and outcomes cannot be ascertained. Thus, in this study, it was impossible to establish whether exposure to pregnancy preceded the outcomes. However, cross-sectional studies are convenient for the purposes of exploration and generation of new hypotheses for further research. Despite the limitations of cross-sectional study design, it was appropriate for this study, since this was a new exploration of the intersections of pregnancy, injection drug use, and sex work.

Since the population of injection drug users in Saint Petersburg is highly stigmatized and largely hidden, and WIDUs are subjected to even greater stigmatization than male IDUs, convenience sampling was necessary as it was not feasible to use a random sampling frame to study this population. Thus, inferring the results of this study onto the population of WIDUs is limited.

Another limitation is that rapid tests do not register acute HIV infections; thus, the prevalence of HIV in the study population might potentially be underestimated.

Selection bias may be present since the majority of study participants were women who use programs targeting injection drug users. These women may have different characteristics when compared to women who do not participate in such programs.

Finally, since injection drug use during pregnancy is highly stigmatized behavior, especially when combined with HIV-positive status,^29^ a response bias might have been introduced among those women who reported not using drugs during pregnancy. As with barriers to seeking HIV care discussed above,^28^ it was thought that women who were actively using drugs would also not seek medical care for their pregnancy. Therefore, to assess the potential size of the response bias, the proportion of women who reported seeking medical care for their pregnancy was compared between women who indicated active drug use and who reported not currently using drugs. It was thought that women who misreported their drug use and were in fact using drugs would also not seek medical care. The results showed that 43 % of current drug users sought care, while 83 % of those who reported not using drugs sought care (*p* < 0.0001). If those classified as non-drug users did in fact use drugs, the expected proportion among the non-active drug users would be closer to that of the active drug users. However, the proportion among the non-active drug users is almost double that of the active users providing some evidence that the response bias is not large.

### Strengths

The sample size of this study is larger than any recent prior study conducted among women injecting drugs in Saint Petersburg during recent years. In addition, the 150 HIV-positive pregnant women with experience of intravenous drug use in this study represent 20 to 30 % of the known number of HIV-positive women who give birth in Saint Petersburg yearly. According to internal statistics of the two maternity houses where mothers with unknown or established HIV-positive status give birth, about 500 births by HIV-positive mothers are given in Saint Petersburg yearly.

An objective assessment of HIV status was determined by the use of rapid test rather than the women reporting their status. The estimate of HIV prevalence in this study might not reflect the actual prevalence among WIDUs, but it might provide HIV status information about the source population and directions for further investigation.

## Conclusions

Findings from this study support the need to urgently develop public health interventions for women injecting drugs. Thirty percent of currently pregnant WIDUs found out about their pregnancies after 12 weeks of gestation, eliminating the opportunity to cease drug use during the first trimester or legally terminate the pregnancy. Other studies conducted in Saint Petersburg have demonstrated that WIDUs need comprehensive intervention programs aimed at primary, secondary, and tertiary prevention of HIV infection;^30^^,^ 31 prevention of unplanned and unwanted pregnancies;^11^^,^ 12 and assistance in changing risk behaviors in pregnant WIDUs.

Strong associations found in this study suggest that the targeted implementation of effective intervention programs for pregnant women using drugs could potentially have a substantial public health impact. In the absence of stable sustainable programs aimed to provide comprehensive social, medical, and drug addiction care for pregnant WIDUs, our study found that pregnancy is still associated with lower prevalence of active drug use and sex work. Thus, one might infer that during pregnancy, the WIDUs’ motivation to change behaviors negatively affecting their health is considerable. The potential effect of targeted programs for pregnant WIDU addressing the prevention of vertical transmission of HIV, providing comprehensive assistance in treating drug addiction, and creating social conditions allowing discontinuing sex work might be significant in terms of improved health status of newborns and mothers and prevention of further spread of HIV and other blood-borne and sexually transmitted infections. The findings that the period of motivation for changing negatively affecting health behaviors might be restricted to the period of pregnancy impose even greater public health significance on potential intervention programs tailored for pregnant WIDUs. Additionally, the finding that HIV-positive women were less likely to actively use drugs during pregnancy compared to HIV-negative women suggests that different types of interventions should be designed for HIV-positive and HIV-negative women.
